# Illustrations of the Theory and Practice of Ventilation, with Remarks on Warming, Exclusive Lightning, and the Communication of Sound

**Published:** 1844-04

**Authors:** 


Art. XII.—*Illustrations of the Theory and Practice of Ventilation, with remarks on warming, exclusive lighting, and the communication of sound.* By D. B. Reid, m.d. f.r.s.e.—*London*, 1844. 8vo, pp. 451.

The eminent chemist who is the author of this book has been so long known to scientific men by his writings and lectures, and by the reputation he has acquired in applying his knowledge practically to the warming and ventilation of many public buildings—more particularly the Houses of Parliament—that the mere announcement of the work is sufficient to ensure its wide circulation. We deem it our duty, nevertheless, to call the special attention of our readers to it: firstly, because it contains much information which cannot fail to be of great use to them in their daily intercourse with the sick; and, secondly, because their acquaintance with its multifarious matter may enable them to be of much service to the community where they reside, by teaching them how to counteract the proceedings of architects and others who, from ignorance of the principles of ventilation and warming, are so constantly committing most injurious blunders in the construction of public and private buildings. No one will read Dr. Reid's volume without being convinced of the vast importance of the subjects of which it treats to the welfare of the public at large, and without acknowledging the debt which society owes to the author for communicating the information so unreservedly, and in a manner so clear and circumstantial as to render it practically available in everybody's hands. Everything is stated in the simplest and most precise form, and every single statement is illustrated by one or more diagrams. In no work, indeed, did we ever see such a profusion of pictorial aids to the understanding of the text; there being, we should think, many more woodcuts than there are pages in the work.

